# Exploring Parents’ Immediate Reactions to Digital Suicide Risk Alerts: Descriptive Study

**DOI:** 10.2196/66349

**Published:** 2025-11-19

**Authors:** Taylor A Burke, Alexandra H Bettis, Nehal Methi, Kathryn R Fox

**Affiliations:** 1 Department of Psychiatry, Massachusetts General Hospital, 185 Cambridge Street, Boston, MA, 02114, United States, 1 617-724-5600; 2 Department of Psychiatry, Harvard Medical School, Boston, MA, United States; 3 Department of Psychiatry & Behavioral Sciences, Vanderbilt University Medical CenterNashville, TN, United States; 4 Department of Psychology, University of Denver, Denver, CO, United States; 5 The Ohio State University College of Medicine, Columbus, OH, United States

**Keywords:** digital monitoring, smartphone app, suicide, self-injury, risk, guardians, parents, youth, risk alerts, youth suicide, monitoring, mHealth, mobile health, apps, applications, smartphones, suicide risk, suicidal ideation, parental perceptions, mobile app, alerts, subscription

## Abstract

**Background:**

Youth suicide is a critical public health crisis. Subscription-based parental digital monitoring apps have emerged to monitor youths’ web-based activities and promptly alert parents in case of detected suicide risk. Parents’ responses to digital suicide risk alerts could significantly influence their children’s immediate and long-term well-being. However, parent experiences receiving these alerts and their impact on parent–child and co-parent relationships remain unclear.

**Objective:**

This study aimed to examine parental perceptions of digital suicide risk alerts, as well as characterize parents’ emotional, cognitive, and behavioral responses to alert receipts, and evaluate the impact on their relationships with their child and co-parent.

**Methods:**

Parents subscribed to the MMGuardian app (Pervasive Group Inc) who received a suicide risk alert were invited to complete a web-based survey. The survey assessed demographics, experiences with emotional, cognitive, and behavioral responses to alerts, and the impact on parent–child and co-parent relationships.

**Results:**

The final sample included 217 parents with an average age of 40.1 (SD 6.82) years and who were predominantly women (183/217, 84.3%), White (189/217, 87.1%), and non-Hispanic (183/217, 84.3%). Approximately 32% (69/217) of parents reported learning for the first time that their child may be at risk for suicide or self-harm through this alert. Parents reported a moderate level of concern about their child’s self-injurious thoughts and behaviors risk based on review of the alert (mean 2.12, SD 0.985). Parents reported experiencing a range of negative emotions in response to receiving a risk alert, with nervousness and sadness being the most common. Self-blame was the most strongly endorsed cognitive response, while thoughts of hopelessness, permanence (eg, “My child is never going to be okay”), and skepticism (eg, “My child is manipulating me”) were less common. Common behavioral actions taken after receiving the alert involved telling their child they loved them, talking with their child to learn more, validating their child’s thoughts and feelings, helping their child use coping skills, and discussing mental health with their child. Approximately 72% (156/217) of parents reported that the alert positively impacted their closeness with their child. Participants were between moderately and quite a bit in sync (mean 2.25, SD 1.26) with their co-guardian regarding alignment in perceptions of their child’s current risk as well as their corresponding thoughts, emotions, and plans related to child risk.

**Conclusions:**

Findings highlight several points for potential intervention to better support parents in navigating responding to their child’s possible suicide risk. As digital parental monitoring tools grow in popularity, research is essential to optimize these tools for the benefit of children and families. Improved understanding of the impact of digital suicide risk alerts will guide the development of effective digital support tools, empowering parents to help both themselves and their child.

## Introduction

### Background

Suicide is a well-established public health crisis, representing the tenth leading cause of death overall in the United States [1], the second leading cause of death for youth aged 10 to 14 years, and the third leading cause of death for youth aged 15 to 24 years [2]. Nonfatal suicidal thoughts and behaviors are even more common; approximately 20% of high school students report that they had seriously considered suicide, and 9% report a suicide attempt in the past [3]. Despite decades of prevention and intervention efforts, however, rates of self-injurious thoughts and behaviors (SITBs), inclusive of nonsuicidal self-injury and suicidal thoughts and behaviors, in youth remain high.

### Self-Injurious Thoughts and Behavior Disclosures

The identification and treatment of SITBs in youth rely on their disclosure. Research suggests that rates of SITB disclosure among youth are variable, with approximately 35% to 43% of youth reporting never disclosing their history of SITBs to their parents or guardians in their lifetime [4-8]. Importantly, youth consistently report disclosing more frequently to friends as compared to trusted adults, including parents, guardians, and caregivers (hereafter “parents”; according to a study by Fox et al [4]). Relatedly, parent and child reports of the child’s SITBs are often discrepant, with parents reporting fewer child SITBs than the child’s self-report [9-11]. Without disclosure, the youth experiencing SITBs may not receive critical parental support and may not access the necessary psychiatric care, as youth often are reliant on parents to identify providers, transport to, and pay for mental health treatment.

### Digital Monitoring of Suicide Risk

In parallel to the growing public health crisis of youth suicide, the widespread use of smartphones and social media has reshaped the lives of youth. An astounding 96% of youth regularly use social media, with nearly half of teens reporting they use social media “almost constantly” [12]; this statistic has doubled since 2015. This has created a substantial opportunity to harness youths’ digital footprints to enhance suicide risk detection on a population-wide scale. Understandably, parents are deeply concerned about safeguarding their children from the potential hazards of digital media engagement. To address these concerns, subscription-based parental digital monitoring apps have emerged. As of 2020, 72% of parents reported using parental control tools to oversee and, in some cases, limit aspects of their children’s smartphone use [13]. The parental digital monitoring app market is currently valued at US$985.12 million, and experts anticipate exponential growth in its worth over the next decade, potentially reaching upwards of US$6 billion by 2029 [14]. In this rapidly expanding market, a growing number of these apps monitor children’s digital traces (eg, text messages, internet searches, and social media activity) to identify suicide-related content shared or accessed and to promptly alert parents to their child’s potential SITB risk via real-time digital suicide risk alerts. While these digital suicide risk alerts require independent validation, which is often hindered by the proprietary nature of these apps, they also have the potential to help identify at-risk youth and aid in SITB prevention, given their already widespread adoption.

### Study Rationale and Aims

Considering the widespread and growing dissemination of tools that digitally alert parents to their children’s suicide risk, it is imperative that we understand the experiences of parents receiving these alerts and how these alerts may impact the parents themselves. In general, poor parental emotion regulation in response to stressors (such as learning of a child’s suicide risk) is associated with poor child emotion regulation and adjustment [15]. Thus, parents’ emotional, cognitive, and behavioral responses to digital suicide risk alerts could significantly impact their children. Although a small body of existing quantitative research delves into the effects on parents of discovering their child’s suicide risk, it is limited by its reliance on retrospective accounts, often with significantly variable or unreported time lapses since discovery [16,17], or limited to small samples [16,18]. To our knowledge, no research has explored the impact on parents of learning about their children’s suicide risk within hours to days after they learned of their children’s risk. Such research is needed to shed light on parents’ more immediate feelings, thoughts, and actions in response to learning about their children’s risk, minimizing the effects of memory and recall biases. The immediate responses of parents could wield a profound influence on their children. Unfortunately, youth often experience SITB disclosures negatively, with youth identifying several family-level (eg, fears of worrying parents) and system-level (eg, fears of hospitalization) barriers to disclosing to their parents [4,19]. Experiencing a disclosure to a parent as aversive, unhelpful, or invalidating may exacerbate youth suicide risk and decrease the likelihood of future disclosures.

To more fully understand how receiving a digital suicide risk alert affects parents, it is also essential to understand how it impacts relationships between caregivers. In a review of existing studies examining experiences of living with a suicidal family member, authors found that marital relationships are often impacted due to disagreements about the right approach to manage child suicide risk and different emotional reactions to the child’s risk [20]. However, it remains unclear how parent relationships may be impacted in cases of digital suicide risk alerts that may reflect more ambiguous suicide risk, especially when considering more diverse family structures and co-parenting situations.

No research to our knowledge has explored the impact on parents of specifically receiving digital suicide risk alerts based on their child’s web-based activity. Research elucidating how parents respond to learning that their child may be at risk for suicide through digital means is critical, given the significant amount of time adolescents spend in the digital world and the expected increased rate at which parents will receive digital suicide risk alerts. Improved understanding of how parents respond to these alerts emotionally, cognitively, and behaviorally may help to shed light on how we can better support parents in these critical moments. There is significant opportunity to leverage this information to create digital support tools to empower parents to help themselves and their child upon digitally learning their child is at suicide risk.

This study has 4 primary aims that seek to explore parents’ experiences receiving digital suicide risk alerts about their child. First, we aim to characterize a sample of parents actively using MMGuardian, a digital monitoring app developed by Pervasive Group Inc, that monitors their child’s web engagement and potential suicide risk, including demographic and clinical characteristics. Second, we aim to examine how parents engage with the digital suicide risk alerts and their degree of concern related to their child’s SITB risk upon receipt of these alerts. Third, we aim to characterize parents’ emotional, cognitive, and behavioral responses in the immediate period after they receive a digital suicide risk alert concerning their child. Finally, we aim to evaluate the impact of the alerts on their relationships with their child and co-parent.

## Methods

### Procedures

MMGuardian is one popular paid phone app of many on the market developed to help parents monitor the safety of children’s digital interactions. MMGuardian generates safety alerts based on a proprietary algorithm that uses AI to identify if the content of a message is indicative of specific high-risk situations. Suicide safety alerts are one such alert that is triggered by this algorithm, which runs continuously on the child’s phone and monitors popular social media apps, web browsing activity, as well as SMS and iMessage text messages. Importantly, because of the proprietary nature of their algorithm, these alerts have not been independently validated, and our research team does not have access to the specifics of how this algorithm operates. Parents are notified of alerts through notifications on their phones from the MMGuardian app or through logging into their MMGuardian app on a computer. MMGuardian has identified over 1.5 million cases of potential imminent suicide risk, demonstrating the potential use of these types of algorithms to identify youth at risk in a scalable manner.

Parents based anywhere in the United States who were currently subscribed to the MMGuardian app and received a digital suicide risk alert from MMGuardian during the study recruitment period were invited to participate in the study via an in-app notification and email invitation. A total of 1484 interested parents were then provided information about the study and prompted to complete a screening survey assessing inclusion criteria (use of MMGuardian, receipt of a suicide safety alert within the past 48 hours, and concern beyond “not concern” upon receiving the most recent suicide safety alert) in Qualtrics; 1463 parents began the screening survey, and of these, 938 parents were eligible and invited to complete an e-consent form to participate in the survey study. If they were eligible and had provided consent, participants were sent a link to a one-time Qualtrics study survey, and they were given 14 days from receipt of the digital suicide risk alert to complete the survey. The final analytic sample included 217 participants.

### Ethical Considerations

Study procedures were approved by the Denver University institutional review board (1824046‐5). All participants provided informed consent via an e-consent form that explained the nature and potential consequences of participating in the study. Participants who completed the survey were compensated for their participation via a US $12 electronic gift card. All participants were provided with extensive suicide-related crisis resources at the start and end of the study. Data were deidentified for analysis and publication.

### Participants

We enrolled 421 participants in the study between December 2022 and June 2023. Of the enrolled participants, 290 participants completed at least 75% of the survey, and of these, 217 answered that they were at least “a little bit” concerned about their child’s suicide or self-harm risk at the time of receiving and reviewing the digital suicide risk alert. MMGuardian intentionally sends suicide safety alerts that are highly sensitive to potential suicide risk. As a result, some alerts may flag content that is ambiguous, taken out of context (eg, slang), or about another person who is not their child. In these cases, parents may quickly determine that the alert does not warrant concern. Given that our study aimed to examine parental responses to perceived suicide risk, we excluded parents who reported no concern at all upon reviewing the alert, as their inclusion could obscure patterns relevant to parents responding to genuine risk. Therefore, the final analytic sample for the current study included 217 parents. Of note, 2-tailed *t* tests revealed there were no differences in child or parent age between those who did and did not report nonzero concern upon viewing the alert. Participants had an average age of 40.1 (SD 6.82) years and were predominantly women (183/217, 84.3%), White (189/217, 87.1%) and non-Hispanic (183/217, 84.3%).

### Measures

#### Demographic Information

Participants reported on their own demographic information, including age, gender, race, ethnicity, annual household income, and educational level. Participants also reported on their child’s demographic information, including child age, race, ethnicity, sex, gender, and sexual orientation.

#### Child Treatment History

Parents reported on whether their child was currently in treatment for any mental health reasons or had ever received mental health treatment in their lifetime.

#### Parent Suicide and Self-Injury History

Participants reported on their history of suicidal thoughts, suicide plans, and suicide attempts using yes or no questions adapted from the Self-Injurious Thoughts and Behaviors Interview–Revised [10]. Participants also reported whether they had a history of treatment for suicidal thoughts or behaviors (yes or no) and whether they had a family history of suicide (yes or no).

Participants also completed a set of measures developed for the current study about the digital suicide risk alert that they received for their child and their responses to receiving the alert. Questions were developed through a multistep process informed by both the extant literature and our own qualitative research. We drew on prior studies that have examined parental responses to learning about youth suicidal and nonsuicidal self-injury risk [16-18]. These studies provided a foundational understanding of emotional, cognitive, and behavioral dimensions of parental reactions. In addition to consulting the extant literature, we generated items based on our team’s qualitative research involving collecting and thematically coding adolescent descriptions of how their parents reacted to learning about their SITBs [4,21].

#### Digital Suicide Risk Alert Information

Parents reported on whether this was their first time receiving an MMGuardian digital suicide risk alert for their child (yes or no). If they had previously received an alert, they were asked to estimate the number of times they have received digital suicide risk alerts for their child. Parents also completed information about the content of the alert, including the platform that triggered the alert (ie, message, internet search, image, or other), and date and time of the alert. Finally, parents reported how long after MMGuardian sent the alert that the parent viewed the alert information (response options included 0‐5 min, 5‐15 min, within 1 h, within 2‐8 h, within 8‐24 h, within 24‐48 h, after 48 h, or “I don’t know”) and how long since learning about the alert they began this study.

#### Parent Knowledge of Child’s SITB Risk

For parents who reported this was not the first time they learned about their child’s potential SITB risk, they were asked to report when they first learned about their child’s potential risk (response options included this week, this month, within the past 12 mo, >1 y, >2 y, 3‐5 y, 6‐10 y, or >10 y). Further, parents were asked if they ever noticed any signs their child may be at risk for suicide or self-harm prior to receiving this digital suicide risk alert.

#### Parent Level of Concern About Their Child’s SITB Risk

Parents were asked the degree to which they were concerned about their child’s suicide or self-harm risk after reviewing the alert information on a scale from 0=not at all to 4=extremely. Parents then specifically rated their degree of concern about suicidal thoughts, suicidal behaviors, and self-harm on a scale from 0=none at all to 4=extremely high.

#### Parent Responses to Alerts

Participants completed items about their emotional, cognitive, and behavioral responses to receiving a digital suicide risk alert about their child. To assess *emotional responses*, participants rated a range of emotion items (eg, nervous, confused, sad, and guilty) on a scale from 0=not at all to 4=extremely. To assess *cognitive responses*, participants rated the degree to which they had specific thoughts (eg, “This is my fault”, “I failed them as a parent/caregiver”, and “We will get through this”*)* immediately after reviewing the alert on a scale from 0=not at all to 4=extremely. To assess *behavioral responses*, participants were given a list of potential behavioral responses and asked to select all that they did after seeing the digital suicide risk alert. Response options included help-seeking behaviors (eg, “Called 911” and “Tried to make an appointment with a new provider to get my child mental health treatment”*), talking* with family and support systems (eg, “Held a family meeting or discussion about it”, “told the other caregiver(s) in the home,” and “notified my child’s teachers”*),* restriction of access to people, places, or things (eg, “Limited the time my child was allowed to use their phone or device” and “Limited the time my child was allowed to spend time alone*”*), and supportive behaviors (eg, “Normalized my child’s thoughts or feelings” and “Told my child that I love them*”*). See Multimedia Appendix 1 for the full measure.

#### Impact of the Digital Suicide Risk Alert on Parent Relationships With Their Child

Participants answered questions about how receipt of the digital suicide risk alert impacted their relationship with their child. Parents rated whether receipt of the alert had a negative impact, no impact, or a positive impact on several aspects of their relationship with their child (eg, closeness with your child, overall relationship with your child, trust in your child, and your child’s trust in you). See Multimedia Appendix 1 for the full measure.

#### Parent and Co-Parent Alignment in Response to and Communication About Alert

Participants completed items assessing their alignment with potential co-parent(s) regarding communication around and response to the digital suicide risk alert. Specifically, to assess communication alignment, parents were asked to share how well they and their co-parent(s) have communicated with each other and with others (other relatives and health care providers) about their child’s suicide or self-harm risk across 4 items on a Likert-type scale. They also rated their level of alignment with co-parents on their level of concern, thoughts, and emotions around their child’s suicide risk across 4 items (Multimedia Appendix 1). These scales showed excellent reliability, with Cronbach α of 0.92 and 0.95, respectively.

### Data Analytic Plan

First, we examined descriptive characteristics of participant reports of their child’s history of mental health treatment, SITB risk, and experiences with digital suicide risk alerts. Second, we examined descriptive characteristics of participants’ engagement with digital suicide risk alerts, including average time until they saw the alert and the level of concern the alert prompted. Third, we examined descriptive characteristics of participants’ emotional, cognitive, and behavioral responses in the immediate period after they receive a digital suicide risk alert. Finally, we examined impacts of this alert on relationship quality with child and co-parent.

## Results

### Aim 1: Demographic and Clinical Characteristics of the Sample

**Figure 1.**
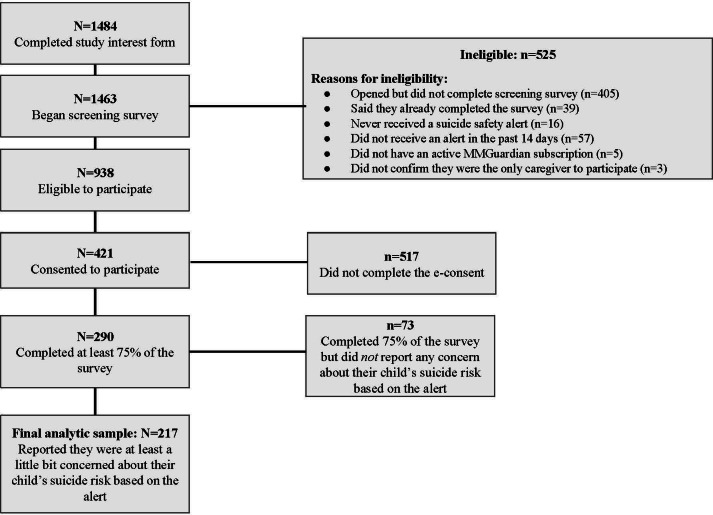
Recruitment flow diagram.

The final analytic sample included 217 parents (Figure 1). As mentioned earlier, the sample was predominantly women who were White and non-Hispanic. A small proportion of the sample was men (30/217, 13.8%), and only 4 participants endorsed a non-cisgender identity. Similarly, there was very limited racial and ethnic diversity among the sample, with a minority of the sample identifying as any other racial category other than White. Participants were primarily the biological parent of the child who triggered the digital suicide risk alert (163/217, 75.1%), and the average household income for participants ranged from US$ 5000 (8/217, 3.7%) to US$150,000 or more (24/217, 11.1%). Parent’s educational background ranged from “some high school” (7/217, 3.3%) to doctorate-level degree (4/217, 1.9%).

The children whose parent participants received the alert about were on average 14.3 (SD 2.1) years of age and were predominantly girls (135/217, 62.2%). Notably, parents reported that 29 (13.4%) children were transgender or gender diverse. Per parent reports, child racial and ethnic identities were similar to those of their parent completing the survey. Table 1 shows the demographic information about the full sample, including parent-reported demographics about their child.

Regarding clinical characteristics of the sample, over half (121/217, 56%) of parent participants reported a personal lifetime history of suicide ideation, with nearly a quarter (47/217, 22%) reporting a lifetime suicide attempt and 77 (33%) reporting they received mental health treatment for SITBs, specifically. In addition, most participants (168/217, 75%) reported that their child had a history of receiving professional mental health treatment or support, with 143 (64%) reporting that their child is currently receiving this care.

**Table 1.** Demographic characteristics of the sample (N=217).

Demographic characteristics	Value
Guardian	
Age (y)	
Mean (SD)	40.1 (6.82)
Missing^a^, n (%)	118 (54.4)
Gender, n (%)	
Agender	2 (0.9)
Man	30 (13.8)
Nonbinary	1 (0.5)
Queer	1 (0.5)
Woman	183 (84.3)
Race, n (%)	
AI^b^ or AN^c^	2 (0.9)
Asian	1 (0.5)
Black	7 (3.2)
Multiracial	9 (4.1)
NH^d^ or PI^e^	1 (0.5)
Other	5 (2.3)
White	189 (87.1)
Prefer not to answer	2 (0.9)
Unsure	1 (0.5)
Ethnicity, n (%)	
Hispanic (Another Hispanic, Latino/a/x, or Spanish origin)	8 (3.7)
Hispanic (Cuban)	1 (0.5)
Hispanic (Mexican, Mexican American, or Chicano/a/x)	13 (6)
Hispanic (multiple origins)	1 (0.5)
Hispanic (Puerto Rican)	5 (2.3)
Non-Hispanic	183 (84.3)
Prefer not to answer	2 (0.9)
Prefer to self-describe	1 (0.5)
Some other race, ethnicity, or origin	1 (0.5)
Missing	2 (0.9)
Annual household income (US $), n (%)	
<5000	8 (3.7)
5000 to 9999	1 (0.5)
10,000 to 14,999	4 (1.8)
15,000 to 25,999	16 (7.4)
26,000 to 49,999	33 (15.2)
50,000 to 74,999	64 (29.5)
75,000 to 99,999	38 (17.5)
100,000 to 149,999	26 (12)
≥150,000	24 (11.1)
Missing	3 (1.4)
Guardian relationship to child, n (%)	
Adoptive parent	18 (8.3)
Aunt or uncle	5 (2.3)
Biological parent	163 (75.1)
Foster parent	1 (0.5)
Grandparent	2 (0.9)
Other adult not listed here	4 (1.8)
Other relative	4 (1.8)
Step-parent	20 (9.2)
Child (based on guardian report)	
Age (y)	
Mean (SD)	14.3 (2.01)
Missing, n (%)	1 (0.5)
Gender (could select multiple), n (%)	
Agender	1 (0.5)
Androgynous	1 (0.5)
Boy or man	53 (24.4)
Child unsure or questioning	6 (2.8)
Girl or woman	135 (62.2)
I am unsure and child unsure	1 (0.5)
Multiple genders selected	5 (2.3)
Nonbinary	9 (4.1)
Queer	1 (0.5)
Unsure or questioning	5 (2.3)
Sexual orientation (could select multiple), n (%)	
Heterosexual	114 (52.5)
Child unsure or questioning	35 (16.1)
Bisexual or pansexual	33 (15.2)
Gay or lesbian or homosexual	10 (4.6)
No label	7 (3.2)
I am unsure	8 (3.7)
Multiple categories selected (eg, heterosexual, gay, and asexual)	4 (1.8)
Queer	2 (0.9)
Prefer not to answer	2 (0.9)
Asexual	2 (0.9)
Race, n (%)	
AI or AN^b^	4 (1.8)
Asian	1 (0.5)
Black	7 (3.2)
Multiracial	19 (8.8)
Other	5 (2.3)
White	177 (81.6)
Prefer not to answer	2 (0.9)
Unsure	2 (0.9)
Ethnicity, n (%)	
Hispanic (Another Hispanic, Latino/a/x, or Spanish origin)	8 (3.7)
Hispanic (Mexican, Mexican American, or Chicano/a/x)	22 (10.1)
Hispanic (multiple origins)	2 (0.9)
Hispanic (Puerto Rican)	6 (2.8)
Non-Hispanic	170 (78.3)
Prefer not to answer	5 (2.3)
Some other race, ethnicity, or origin	3 (1.4)
Missing	1 (0.5)

a Due to a coding error, a question assessing parent or guardian age was not added until about half-way through study recruitment, so only 99 participants provided parent age information.

b AI = American Indian.

c AN: Alaska Native.

d NH: Native Hawaiian.

e PI: Pacific Islander.

### Aim 2. Characterize Parent Engagement With and Level of Concern Related to the Digital Suicide Risk Alert

Notably, 32% (69/217) of participants reported that this was their first time learning that their child may be at risk for suicide or self-harm, with 42% (91/217) reporting that this was the first time that they had ever received a digital suicide risk alert through MMGuardian for their child; 23% (50/217) of participants reported that this was both their first digital suicide risk alert *and* the first time that they learned their child may be at risk for suicide or self-harm generally. Most participants (151/217, 73.3%) viewed the digital suicide risk alert within 5 minutes of receiving the alert from MMGuardian (Table 2), and 86.7% (188/217) of participants completed the study survey within 2 days of receiving a digital suicide risk alert. Just over half of participants reported that they began participating in this study the same day that they saw the alert (115/217, 53%); 22.6% (49/217) reported that they started the survey within 2 days of reviewing the alert and 8.3% (18/217) started 3 days after receiving the alert; remaining participants (23/217, 10.6%) reported that they started within 4 and 14 days of receiving the safety alert while 2.3% (5/217) reported “other.” Parents were on average somewhat concerned about their child’s level of SITB risk at the time of receiving the alert (mean 2.12, SD 0.985; range 1‐4). On average, participants reported having received an average of 14.94 (SD 26.79; range 0‐200) digital suicide risk alerts from MMGuardian since subscribing.

**Table 2.** Descriptive characteristics of suicide risk alert and parent report of child suicide risk history (N=217).

Characteristics	Value
Time passed until viewed alert, n (%)	
After 48 hours	2 (0.9)
I don’t know	6 (2.8)
Within 0‐5 minutes	151 (69.6)
Within 1 hour	13 (6)
Within 10‐15 minutes	27 (12.4)
Within 2‐8 hours	8 (3.7)
Within 8‐24 hours	6 (2.8)
Within 24‐48 hours	3 (1.4)
Missing	1 (0.5)
First learning of child suicide risk, n (%)	
No	147 (67.7)
Yes	69 (31.8)
Missing	1 (0.5)
First MMGuardian digital suicide risk alert, n (%)	
No	126 (58.1)
Yes	91 (41.9)
Perceived risk immediately after viewing alert, mean (SD; range 0‐4)	
Nonsuicidal self-injury	1.42 (1.18)
Suicide ideation	1.65 (1.21)
Suicide attempt	0.77 (0.97)
Level of concern immediately after viewing alert	
Mean (SD; range 1-4)	2.12 (0.985)
Median (min, max; range 1-4)	2.00 (1.00, 4.00)

### Aim 3: Characterize Parent Emotional, Cognitive, and Behavioral Responses After Receiving a Digital Suicide Risk Alert

Mean parent emotional and cognitive responses (rated on a scale from 0 = not at all to 4=extremely) are shown in Table 3. The most strongly reported feelings were nervous, sad, stupid, confused, and guilty. The most strongly endorsed thoughts were “We’ll get through this,” “I am responsible,” “This is common,” “I wish I’d acted differently,” and “I should’ve acted differently.”

**Table 3.** Thoughts and feelings endorsed after receiving a suicide safety alert.

Thoughts and feelings after receiving an alert	Rating, mean (SD)
Thoughts	
This is my fault	0.9 (1.19)
I am responsible	2.5 (1.34)
I haven’t done enough	1.4 (1.31)
I failed	1.18 (1.31)
I should’ve acted differently	1.47 (1.31)
I wish I’d acted differently	1.53 (1.33)
Helpless	1.38 (1.34)
Hopeless	0.66 (1.01)
My child will never be ok	0.67 (1.04)
This is common	1.78 (1.17)
We’ll get through this	2.92 (1.16)
This is a phase	1.08 (1.16)
My child wants attention	0.63 (1.01)
My child is manipulating	0.52 (0.98)
This doesn’t make sense	0.64 (1.02)
Feelings	
Nervous	2.05 (1.2)
Confused	1.38 (1.21)
Stupid	1.42 (1.23)
Relieved	0.46 (0.97)
Angry	0.56 (0.94)
Frustrated	0.99 (1.14)
Annoyed	0.47 (0.86)
Sad	1.69 (1.28)
Guilty	1.31 (1.31)
Ashamed	0.58 (1.04)
Suspicious	0.68 (1.03)
Wary	0.91 (1.12)

### Behavioral Responses

**Figure 2.**
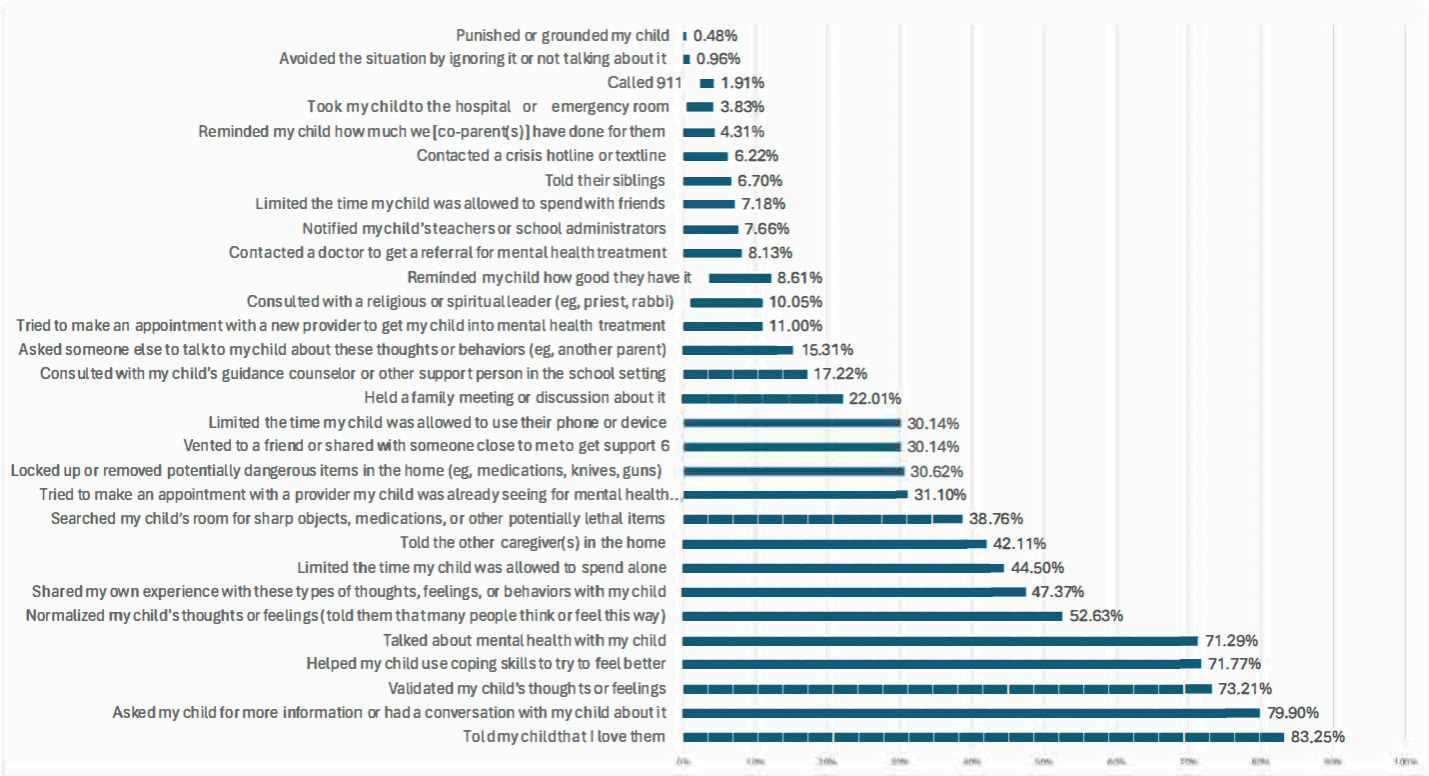
Parent behavioral responses.

Parents described a wide range of behavioral actions taken after receiving the digital suicide risk alert (Figure 2). Most commonly, these involved telling their child they loved them, talking with their child to learn more, validating their child’s thoughts and feelings, helping their child use coping skills, and talking about mental health with their child. Least commonly endorsed were punishing or grounding their child, avoiding the situation by ignoring it or not talking about it, and calling 911 or going to a hospital emergency room for immediate support.

### Aim 4: Examine the Impact of the Digital Suicide Risk Alert on Parent Relationships With Their Child and Co-Parent

**Figure 3.**
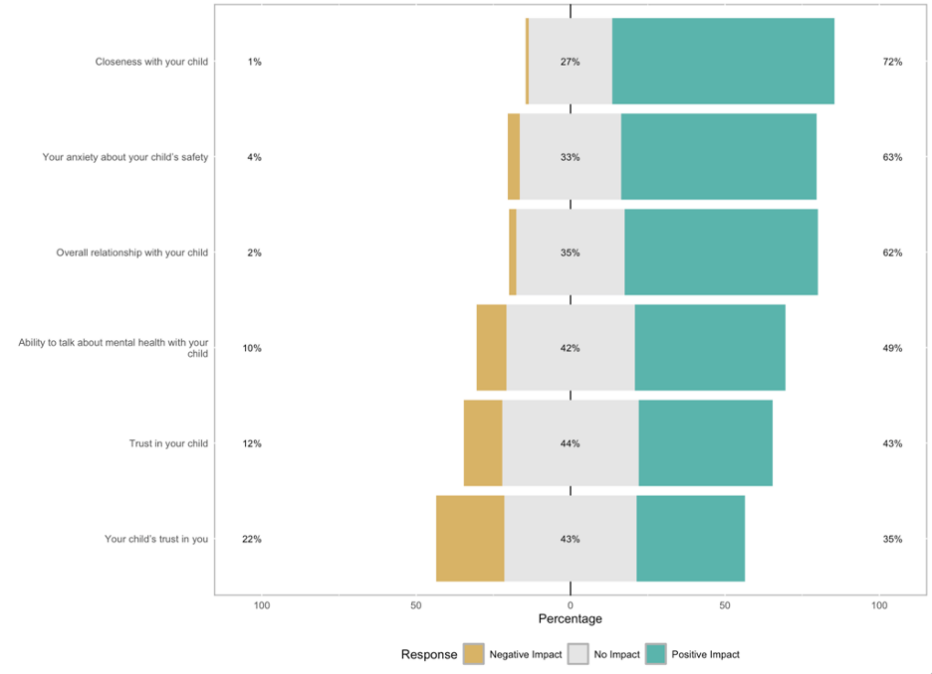
Reported impact of alerts on relationship with child.

Next, we examined whether parents reported that they experienced a negative impact, no impact, or a positive impact on several aspects of their relationship with their child. Descriptive statistics across all items are shown in Figure 3. Parents indicated that these alerts had a largely positive impact on their relationships with their child across domains. However, a notable proportion of parents reported negative impacts specifically on their child’s trust in them after receiving the alert.

Finally, we examined the degree to which co-parents aligned in their perceptions of their child’s risk and response to that potential risk among those parents that reported they share caretaking responsibilities with another person (186, 85.7% parents of the full sample). Regarding alignment in perceptions of their child’s current risk as well as their corresponding thoughts, emotions, and plans related to child risk, parents reported an average score of 2.25 (SD 1.26), indicating that they were between moderately and quite a bit in sync. Notably, 18.5% (34/186) of participants reported an average score of 1 or lower (ie, little to no alignment) across items assessing co-parent alignment in perceptions of their child’s current risk as well as their corresponding thoughts, emotions, and plans related to child risk. Similarly, regarding communications about their child’s SITB risk, parents reported an average score of 2.40 (SD 1.26), again indicating that they have done between moderately and quite a bit well in terms of communication with each other, their child, and others (eg, mental health providers). Further, 24.6% (46/186) reported an average score of 1 or lower across items assessing alignment in communications about their child’s SITB risk.

## Discussion

### Principal Findings

The frequency of parents receiving digital suicide risk alerts from app-based subscriptions like MMGuardian is already high, and use of these apps is anticipated to increase exponentially in the near future. Despite this growing trend, it is unclear how parents react to receiving these alerts. In addition, there is limited research delving into parents’ *immediate* emotional, cognitive, and behavioral responses to learning about their children’s suicide risk, regardless of how they learn about the risk. This study addresses these gaps by capturing parents’ immediate responses to digital suicide risk alerts and providing valuable insights into the specific domains in which they may require support, with the goal of ultimately enhancing the support provided to their at-risk children.

The first aim of this study was to characterize the sample of parents who receive suicide risk alerts about their child via a digital monitoring app that monitors their child’s web-based engagement and potential suicide risk. Results indicate that our sample was largely demographically homogeneous, predominantly comprising White, non-Hispanic biological mothers. Although the demographic characteristics of our sample are congruent with the population commonly encountered in mental health treatment settings, the degree of homogeneity within our sample is notably more pronounced. Aside from race and gender, there are other aspects of this sample that are unique. Over half of the parents in our sample reported a lifetime history of suicidal ideation and nearly a quarter reported a history of suicide attempt. This suggests that many parents in our sample have experienced significant mental health distress and may have a history of mental health treatment themselves. Several factors may explain this elevation, including that parents with a SITB history may have been more likely to participate in our study and that SITB risk may be accounted for in part by genetic influences [22]. Though not assessed directly, given many parents themselves had self-reported SITB histories, it is possible this influenced parents’ decision to subscribe to MMGuardian for their child; it is also possible that their child’s web-based activities and mental health led to their interest in using a digital monitoring app. Motivations behind parents’ use of these apps are an important question for future research in this area.

Interestingly, about a third of parents reported that the digital alert was the first time they had ever become aware of their child’s potential suicide risk. This significant proportion underscores the potential impact these apps may have as a critical initial point of awareness for parents regarding their child’s risk for SITBs. Learning about a child’s SITB risk through an app may be more isolating than learning it from a mental health care provider who can offer direct support and resources. However, if these apps were equipped with detailed, evidence-based information on how to support parents and provide them with skills to effectively discuss and manage their children’s risk, they could be highly beneficial. Many parents may benefit from evidence-based guidance on assessing and managing their child’s SITB risk, especially those learning of the potential risk for the first time as they may be at a particularly pivotal juncture. Further, given the increased use of digital monitoring apps more broadly, more and more parents could learn about their child’s SITBs for the first time through a notification on their phones rather than from a mental health provider or in a traditional context (eg, by a mental health provider). Educating parents on how to have these initial conversations could set the stage for how children communicate with their parents about their SITBs in the future, potentially establishing a positive trajectory for open and supportive dialogue. In addition, most parents viewed the alert within just a few minutes of it being delivered to their phone, which would allow them to respond quickly to their child if actual suicide risk is present, highlighting further the need to offer supports to parents to navigate the aftermath of risk alerts.

Parents reported experiencing a range of negative emotions in response to receiving a risk alert, with nervousness and sadness emerging as the strongest endorsed emotions. Despite this study reporting on parents’ responses to more ambiguous suicide risk, these emotional responses largely parallel emotional responses observed after their child’s suicide attempt; prior studies show that parents commonly report feelings of sadness, anxiety, and hostility [18], as well as guilt, fear, and overwhelm [17].

In line with well-established cognitive-behavioral frameworks, these emotions are likely related to parents’ commonly reported thoughts in this study. Parents strongly endorsed self-blaming thoughts (eg, “This is my fault” and “I should have done something differently”). Such thoughts may lead to emotional experiences of distress, guilt, and shame. In addition, although less common, parents reported thoughts related to hopelessness and permanence (eg, “My child is never going to be okay”) and a number of parents endorsed “skeptical” thoughts (eg, “My child is manipulating me,” “My child just wants attention,” and “My child has it so good, it makes no sense they’re feeling this way”). These cognitions are likely to be associated with the aforementioned “cynical and irritated” emotions. Results of this and prior work highlight that a range of negative thoughts and emotions are common after learning about child suicide risk, that adolescent fears about scaring parents or having parents respond skeptically to their SITB [4] are not entirely unfounded, and that adolescent perceptions of their parents' affective responses to learning about their SITB risk include some of the same emotions (eg, sadness, anger, and surprise [6]).

Altogether, it is important to consider that these parental affective and cognitive responses can be both normative *and* important to address to best support youth who might be at risk for suicide. Indeed, prior research demonstrates parents’ ability to regulate emotions is directly associated with their child’s emotional experiences and regulation [15,23]. Results highlight the importance of teaching parents emotion regulation skills that they can apply when learning about child suicide risk and how to respond in ways that are supportive and validating, even amid emotions and thoughts characterized by self-blame, confusion, or skepticism.

Our results also suggest that parents frequently engage in a wide range of behaviors in the immediate aftermath of receiving alerts that are supported in the literature as promoting child well-being and are associated with reduced SITBs–validation, emotional support, open communication about mental health, and supporting positive coping [23-25]. Also encouraging, a significant proportion of parents reported responding to these risk alerts by directly asking their child about their self-harming (nonsuicidal self-injury; 74%) and suicidal thoughts (76%). Importantly, a failure to directly ask these questions hampers parents’ ability to gauge the severity of risk, make informed decisions regarding the necessary level of care, and may even contribute to the further stigmatization of these experiences. Barriers to asking SITB questions directly may include not knowing what or how to ask, as well as holding the erroneous belief that asking directly about SITBs may inadvertently instigate these thoughts and behaviors [26]. While promising that most parents did initiate these conversations, that one quarter of parents in the present sample reported they did not directly ask about these experiences, despite feeling concerned about their child’s risk, highlights the potential use of psychoeducation interventions aimed at reducing barriers to direct inquiry.

In response to receiving a suicide risk alert, nearly half of parents reported limiting the amount of time their child spent alone. A growing body of evidence indicates that increased parental monitoring may be protective against SITB risk in youth. In a birth cohort study of over 6000 youths, increased parental monitoring predicted reduced risk of self-harm [27] and improved trajectory of suicidal ideation during treatment over a 12-month period [28]. Furthermore, less parental monitoring is associated with increased odds of adolescent NSSI onset over a 1-year follow-up [29]. In the 2021 CDC Youth Risk Behavior Survey of over 17,000 US high school students, youth reporting high levels of perceived parental monitoring reported fewer suicide attempts compared to youth reporting low levels of parental monitoring [30]. Importantly, parent-focused interventions can successfully increase parent monitoring of child risk behaviors [31-33] and may be a helpful tool to digitally provide parents after receiving suicide risk alerts.

Many parents indicated limiting child access to things or people in response to learning about their child’s potential risk. We found that about a third of parents engaged in lethal means restriction (eg, searching for potentially lethal items in the home and locking them up). When risk is indicated, lethal means restriction is an effective tool to reduce suicide risk [34]. Research supporting the use of means restriction in youth is promising, with evidence indicating that parents are critical to the enactment of means restriction in the home [35-39]. At the same time, nearly a third of parents responded to risk alert receipt by limiting child access to their phone or device and just under 10% by reducing the amount of time their child can spend with friends. Although in some cases, these responses may be needed to maintain adolescent safety, it is important to consider how such responses may be perceived as a punishment for SITB risk in ways that may reduce future SITB disclosures among youth [4]. In addition, a small percentage (4.3%‐8.6%) reported responding by “reminding” their child how good they have it or how much their parents or guardians have done for them. Such responses may inadvertently invalidate child feelings and thoughts, ignoring that they may be facing serious stressors or feelings that parents do not fully understand or have not observed.

The final aim in our study was to evaluate parents’ perception of how receiving suicide risk alerts impacted their relationship with their child and co-parent. Parents indicated that these alerts had a largely positive impact on their relationships with their child across domains. It is very promising to see that almost all parents in this study felt that receiving the alerts had a neutral (76/217, 35%) or positive impact (135/217, 62%) on their relationships with their children. That so many parents felt more knowledge about their child’s risk led to improved relationships is significant; future research should explore the reasons why parents felt that alert receipt led to improved relationship quality. It is possible that receiving the alert led to validating and supportive conversations between parent and child, which increased relationship intimacy, though this hypothesis needs to be directly tested.

It is important to also recognize that research shows many youth do not experience suicide risk disclosures to their parents as going well or leading to improved relationship quality [6]. Moreover, parents who participated in this study were likely those who are most interested in mental health in general, given our study advertisements and aims. Thus, it is important to consider these findings alongside the limitation that our study obtained parent-report only, and youth may have different experiences.

About a quarter of parents reported negative impacts on their child’s trust in them. Youth may perceive their parent’s receipt of a suicide risk alert, generated by the use of a monitoring app, as an invasion of privacy. This perception may erode youth trust in their parents, particularly if the child was not adequately informed about the extent to which the app would monitor their digital interactions and notify their parents about potentially concerning engagement. Preserving youth trust in parents is very important in the context of suicide risk prevention; indeed, connecting a child to mental health care is typically contingent on youth disclosure. If a child’s trust in a parent is eroded, the child may be less likely to come forward to their parents in times of need, thereby hindering potentially lifesaving support and intervention. Recent work has begun to explore the impact of parent monitoring of social media and other digital activity on youth mental health outcomes, finding that parents may exhibit patterns of openness and autonomy-supporting or patterns of overcontrol [40]. Given the growing relevance of these digital tools, research examining the role of using digital monitoring as a parenting tool and its associations with youth outcomes, including SITBs, parent-child communication, and trust, is needed.

### Limitations, Strengths, and Future Directions

While the present findings offer unique insight into the experiences of parents in the immediate aftermath of receiving digital suicide risk alerts, some important limitations impact interpretation. First and foremost, the very large majority of participants were White, non-Hispanic women. While this may be reflective of the demographic of parents using digital monitoring services that require monthly subscription costs, our ability to generalize findings to parents of diverse identities or those learning about their child’s risk through other means may be limited. Of note, in a qualitative study comparing White and Black mothers’ responses to learning about their child’s suicide attempt, most emotional distress responses were similar (eg, fear, worry, anger, and guilt); however, there were some notable differences (eg, more reports of disbelief or skepticism about suicide attempts and more focus on coping with distress among Black mothers) [41].

Second, this study was a single-time point, single-perspective investigation into parent responses immediately after learning about child SITB risk, with questions assessing impacts on relationships with child and co-parent that may not have yet been observable and that were missed by focusing solely on a single parents’ perspective. Future dyadic or familial longitudinal studies should aim to characterize the impact of digital suicide risk alert receipt on family relationships and child mental health outcomes as well as future suicide risk. In particular, these digital monitoring apps are new, and research on child or adolescent perceptions of these apps is limited. It will be crucial for future research to explore how this technology affects youth, with an eye toward preventing unintended negative consequences (eg, lowering parent–child trust). Relatedly, it will also be important for future research to examine how “false positives” (ie, alerts sent in the absence of any true child risk) impact family dynamics and youth. For example, do such false positives open or close the door to future conversations about SITBs if that child goes on to experience SITBs for the first time in the future? What patterns of parental responses are associated with greater disclosure later on? It will be important to assess the impact of receiving numerous suicide alerts and whether these repeated alerts index greater child suicide risk.

Another limitation of this study is that we only included parents who reported at least some level of concern about their child’s SITB risk after reviewing the alert. While this approach ensured that parent participants found the alerts relevant, it excluded a subset of parents who may have dismissed the alerts or perceived them as unimportant. Understanding this subgroup is an important area for future research, as they may be more representative of parents who receive this type of alert; given a lack of data on the validity or usefulness of these alerts, it remains unclear whether concern is warranted. At the same time, parents in this study represent parents who may be more sensitive to, and anxious about, their children’s risk for a range of outcomes, including suicide. This may mean that parent reactions in this study are not representative of parental reactions to learning about child suicide risks in a range of other settings. Furthermore, the average number of suicide safety alerts self-reported by parents was high, at nearly 15. It is possible that repeated alerts could impact parents’ emotional and cognitive responses over time, potentially leading to increased vigilance and concern or, conversely, to habituation and reduced concern over time. Therefore, future research should explore the extent to which higher alert frequency impacts parental distress and responsiveness.

Furthermore, questions about emotional and cognitive responses to these alerts were primarily focused on negative or maladaptive emotional and cognitive responses. Future work is needed to examine how more positive cognitions or emotions may arise in response to these alerts.

In addition, MMGuardian’s proprietary algorithm has not been independently validated in the peer-reviewed literature. This limitation is not unique to MMGuardian and, to our knowledge, applies to all digital alerting systems emerging in smartphone and school device algorithm risk alerting systems. Nevertheless, these suicide risk detection systems are commonly relied upon by parents and schools to guide risk management. Similarly, even our validated risk algorithms are imperfect predictors of suicide risk, particularly in the short term. While the lack of formal validation is an important limitation to this work, this study adds important insight into the way parents experience suicide-related alerts from these widely available commercial tools.

Finally, because this study was primarily exploratory and descriptive in nature, sample size calculations were not performed. Instead, sample size was primarily determined by participant interest and funding limitations. It remains unclear whether and the degree to which results would replicate in a new sample, and future hypothesis-driven work with a priori power analysis is needed.

Despite these limitations, this study has several important strengths that set the stage for future work in this area. To our knowledge, this is the first study to examine parents’ nearly immediate responses to receiving a digital alert about their child’s suicide risk. It is also the largest sample characterizing parents’ near-immediate responses to learning about their children’s suicide risk in general. Further strengthening our findings, we believe results are likely to generalize to the growing number of parental monitoring apps that passively monitor children’s web-based activities for signs of suicide risk. However, future research should examine this directly, as these apps have differences that could impact parental responses (eg, levels of sensitivity and type and amount of content monitored).

### Conclusions

In summary, we examined the experiences of parents after receiving a digital risk alert about their child’s potential SITB risk in a sample of 217 parents subscribed to a digital monitoring app. Parent responses and experiences highlight several points for potential intervention to better support parents in navigating learning about and responding to their child’s possible SITB risk. Given the growing popularity of such monitoring tools, research is needed to best understand how to leverage these tools in ways that will most benefit children and families.

## Supplementary material

10.2196/66349 Multimedia Appendix 1 Study measures.

## Abbreviations

SITB: self-injurious thoughts and behaviors
