# Supplementary material for: Exploring Parents’ Immediate Reactions to Digital Suicide Risk Alerts: Descriptive Study
Source: J Med Internet Res. 2025 Nov 19;27:e66349. doi: 10.2196/66349 (PMC12629623; doi:10.2196/66349)
Supplement: Multimedia Appendix 1 [file jmir-v27-e66349-s001.docx]

**Supplemental Materials**

**Study Measures**

1. **Parent level of concern about their child’s SITB risk**

**Immediately after** you received the risk alert and read the information associated with the alert, how concerned were you about your child's suicide or self-harm risk?

o Not at all

o A little bit

o Somewhat

o Very much

o Extremely

1. **Parent responses to receiving the alert**

**Immediately after** you received the alert about your child’s suicide/self-harm risk, to what extent did you think the following?

|  | Very slightly or not at all | A little bit | Moderately | Quite a bit | Extremely |
| --- | --- | --- | --- | --- | --- |
| This is my fault | o | o | o | o | o |
| I am responsible for my child’s mental health | o | o | o | o | o |
| I haven’t done enough as their parent/caregiver | o | o | o | o | o |
| I failed them as a parent/caregiver | o | o | o | o | o |
| I should have done something differently as their parent/caregiver | o | o | o | o | o |
| I wish I had done things differently as their parent/caregiver | o | o | o | o | o |
| I feel helpless as their parent/caregiver | o | o | o | o | o |
| My child is never going to get better | o | o | o | o | o |
| My child is never going to be okay | o | o | o | o | o |
| This happens to a lot of children | o | o | o | o | o |
| We will get through this | o | o | o | o | o |
| This is just a phase | o | o | o | o | o |
| My child just wants attention | o | o | o | o | o |
| My child is manipulating me | o | o | o | o | o |
| My child has it so good, it makes no sense they’re feeling this way | o | o | o | o | o |

**Immediately after** you received this Suicide Safety Alert, how much did you feel the following emotions?

|  | Very slightly or not at all | A little bit | Moderately | Quite a bit | Extremely |
| --- | --- | --- | --- | --- | --- |
| Nervous, worried, or scared | o | o | o | o | o |
| Confused | o | o | o | o | o |
| Surprised | o | o | o | o | o |
| Relieved | o | o | o | o | o |
| Angry or mad | o | o | o | o | o |
| Frustrated | o | o | o | o | o |
| Annoyed | o | o | o | o | o |
| Sad or down | o | o | o | o | o |
| Guilty | o | o | o | o | o |
| Ashamed | o | o | o | o | o |
| Doubtful or suspicious | o | o | o | o | o |
| Wary | o | o | o | o | o |
| Tired | o | o | o | o | o |
| Overwhelmed | o | o | o | o | o |

Since seeing the Suicide Safety Alert: what did you (or your child’s other caregiver(s)) **DO**?

Check all that apply.

Remember: There is no one right way to respond. We just want to better understand how you responded in the moment.

▢ Took my child to hospital/emergency room

▢ Called 911

▢ Contacted a crisis hotline or textline

▢ Tried to make an appointment with a provider my child was already seeing for mental health treatment

▢ Tried to make an appointment with a new provider to get my child into mental health treatment

▢ Contacted a doctor to get a referral for mental health treatment

▢ Asked someone else to talk to my child about these thoughts or behaviors (e.g. another parent, sibling, extended family, one of their friends)

▢ Told their siblings

▢ Told the other caregiver(s) in the home

▢ Held a family meeting or discussion about it

▢ Notified my child’s teachers or school administrators

▢ Consulted with my child’s guidance counselor or other support person in the school setting

▢ Vented to a friend or shared with someone close to me to get support

▢ Consulted with a religious or spiritual leader (e.g., priest, rabbi)

▢ Limited the time my child was allowed to use their phone or device

▢ Limited the time my child was allowed to spend with friends

▢ Limited the time my child was allowed to spend alone

▢ Searched my child’s room for sharp objects, medications, or other potentially lethal items

▢ Locked up or removed potentially dangerous items in the home (e.g. medications, knives, guns)

▢ Punished or grounded my child

▢ Helped my child use coping skills to try to feel better

▢ Validated my child’s thoughts or feelings

▢ Normalized my child’s thoughts or feelings (told them that many people think or feel this way)

▢ Shared my own experience with these types of thoughts, feelings, or behaviors with my child

▢ Talked about mental health with my child

▢ Asked my child for more information or had a conversation with my child about it

▢ Reminded my child how good they have it

▢ Reminded my child how much we [co-parent(s)] have done for them

▢ Told my child that I love them

▢ Avoided the situation by ignoring it or not talking about it

1. **Impact of the digital suicide risk alert on parent relationships with their child**.

How did receiving the Suicide Safety Alert from MMGuardian about your child, [XXX], impact your relationship with your child?

[Response options: Positive impact, no impact, negative impact]

1. Overall relationship with your child
2. Trust in your child
3. Your child’s trust in you
4. Ability to talk about mental health with your child
5. Closeness with your child
6. Your anxiety about your child’s safety
7. Your own mental health
8. **Parent and co-parent alignment in response to and communication about alert.**

Since receiving the alert, how in sync and aligned have you and your co-parent(s) been in your…

|  | Very slightly or not at all | A little bit | Moderately | Quite a bit | Extremely |
| --- | --- | --- | --- | --- | --- |
| Level of concern about your child’s suicide/self-harm risk | o | o | o | o | o |
| Thoughts about the causes of or reasons for your child’s suicide/self-harm risk | o | o | o | o | o |
| Emotions about your child’s suicide/self-harm risk | o | o | o | o | o |
| Plans to respond to your child’s suicide/self-harm risk | o | o | o | o | o |

coparent_comm How well do you think you and your co-parent(s) have…

|  | Very slightly or not at all | A little bit | Moderately | Quite a bit | Extremely |
| --- | --- | --- | --- | --- | --- |
| Communicated with each other about your child’s suicide/self-harm risk | o | o | o | o | o |
| Been on the same page when communicating with your child about their suicide/self-harm risk | o | o | o | o | o |
| Aligned in expressing your concern about your child’s suicide/self-harm risk | o | o | o | o | o |
| Communicated with others (e.g., other relatives, healthcare providers) about your child’s suicide/self-harm risk | o | o | o | o | o |

|  |
| --- |
